# The Peer Reviewers' Openness Initiative: incentivizing open research practices through peer review

**DOI:** 10.1098/rsos.150547

**Published:** 2016-01-13

**Authors:** Richard D. Morey, Christopher D. Chambers, Peter J. Etchells, Christine R. Harris, Rink Hoekstra, Daniël Lakens, Stephan Lewandowsky, Candice Coker Morey, Daniel P. Newman, Felix D. Schönbrodt, Wolf Vanpaemel, Eric-Jan Wagenmakers, Rolf A. Zwaan

**Affiliations:** 1 Cardiff University, Cardiff, UK; 2 Bath Spa University, Bath, UK; 3 University of California, San Diego, CA, USA; 4 University of Groningen, Groningen, The Netherlands; 5 Eindhoven University of Technology, Eindhoven, The Netherlands; 6 University of Bristol, Bristol, UK; 7 University of Western Australia, Perth, Australia; 8 University of Edinburgh, Edinburgh, UK; 9 Monash University, Melbourne, Australia; 10 Ludwig-Maximilians-Universität München, Munich, Germany; 11 KU Leuven, Leuven, Belgium; 12 University of Amsterdam, Amsterdam, The Netherlands; 13 Erasmus University Rotterdam, Rotterdam, The Netherlands

**Keywords:** science, transparency, open research, peer review

## Abstract

Openness is one of the central values of science. Open scientific practices such as sharing data, materials and analysis scripts alongside published articles have many benefits, including easier replication and extension studies, increased availability of data for theory-building and meta-analysis, and increased possibility of review and collaboration even after a paper has been published. Although modern information technology makes sharing easier than ever before, uptake of open practices had been slow. We suggest this might be in part due to a social dilemma arising from misaligned incentives and propose a specific, concrete mechanism—reviewers withholding comprehensive review—to achieve the goal of creating the expectation of open practices as a matter of scientific principle.

## 1. Introduction

Openness and transparency are core values of science [1]. The American National Academy of Sciences Committee on Science, Engineering and Public Policy [2, p. 9] says unambiguously that researchers have a ‘fundamental obligation’ to keep quality records of their research, and that once it is published, that other researchers ‘must have access to the data and research materials’. This responsibility flows from several sources: our epistemic responsibility to substantiate our claims with evidence, our responsibility to the community of scientists from whom we obtained most of our knowledge, and our responsibility to society at large that supports our research. The Royal Society Science Policy Centre [3, p. 38] states that ‘[g]ood [scientific] communication is assessable communication, which allows those who follow it not only to understand what is claimed, but also to assess the reasoning and evidence behind the claim’.

The Committee on Responsibilities of Authorship in the Biological Sciences, National Research Council [4, p. 4] placed the responsibility for open standards on every member of the research community:

[T]he act of publishing is a quid pro quo in which authors receive credit and acknowledgment in exchange for disclosure of their scientific findings. An author's obligation is not only to release data and materials to enable others to verify or replicate published findings (as journals already implicitly or explicitly require) but also to provide them in a form on which other scientists can build with further research. All members of the scientific community—whether working in academia, government, or a commercial enterprise—have equal responsibility for upholding community standards as participants in the publication system, and all should be equally able to derive benefits from it.

For many decades, openness was frustrated by technological limitations. Physical, paper journals simply could not accommodate a more complete record of the methods and data presented in scientific articles. With the advent of digital information technologies and the Internet, these limitations have disappeared. Instead of being stored in dusty boxes in a laboratory, records of one's materials, data, and analyses can be made publicly available for anyone in the world to access indefinitely. For most research—particularly research in the behavioral and social sciences—it is possible to provide a nearly complete public record of the research from consent form templates, surveys, stimuli and experimental instructions and software, to the data and analysis scripts. Over the past few decades, valuable tools have been developed to facilitate open research: data journals (e.g. [5]) and data repositories (e.g. figshare, Data Dryad, Open Science Framework) allow easy storage and organization of research products; many statistical software packages allow complete records of analyses to be kept and shared; projects such as Sweave [6] and Rmarkdown [7] allow the entire process of data cleaning, analysis and document preparation to be combined in a single, reproducible document.

The possibilities allowed by new technological developments are exciting. Scientific progress is accelerated as more data are available for verification, theory-building and meta-analysis, and experimental materials are available for easier replications and extension studies. The availability of stimuli, questionnaires, programmed paradigms and data analysis scripts will make scientific research more efficient, as research can be more easily understood and replicated, mistakes can be found more readily, and needless duplication of effort across laboratories can be reduced. With open research, scientific articles become part of a collaborative effort to learn what is likely to be true, rather than static records of the results of specific analyses. The meaning of a scientific article is not limited to the interpretation that the original authors give it; with open data, the interpretation of experimental data can evolve as theories develop, or as researchers with varying perspectives analyse them. The open scientific future we glimpse on the horizon holds great promise.

And yet, despite the fact that openness in research is a core scientific value—and despite the fact that nearly complete openness in research has never been easier to achieve—open research practices are rare in many parts of the scientific literature. For decades, authors have found low rates of data sharing, across many scientific disciplines [8–15]. We know less about researchers' willingness to share other aspects of their research such as materials, but the practice seems to be relatively rare. Current research practices are not improving drastically, despite the strong arguments in favour of open practices [16].

Although openness is an ethical obligation of scientists that carries many demonstrable benefits, and despite it being easier than ever before due to the availability software and Internet resources, many areas of research are still largely not open. The current approach to advocating openness—a mixture of extolling the benefits of openness to researchers and pushing for policy change at journals and granting agencies—has proved to be insufficient to motivate the adoption of open research practices.

Perhaps the lack of success of this approach is no surprise. The way that the incentives in science are structured, the ability to publish is rewarded above all else. Learning open practices is not difficult, but implementing them does take time and may delay the publication process, even if only for a few days. Opening one's own data and methods could also be perceived as benefiting one's competitors with no guarantee of reciprocal openness, possibly placing one at a disadvantage within one's scientific field. This creates a social dilemma: individuals might value open research practices as being in the best interest of the scientific community, yet simultaneously avoid them because they do not believe that implementing open practices is in their individual best interest. Given this social dilemma, the current slow pace of change is perhaps not surprising.

We believe many researchers are excited by the promise of open scientific practice, and surveys have shown their agreement with open principles [17,1]. Researchers simply need an incentive, and may even welcome a strong incentive if it were offered. We thus believe a change of strategy is needed. The strategy we propose here uses the role of the reviewer as an instrument of change. As a group, reviewers share the power to ensure that articles meet minimum scientific quality standards. What is needed is an affirmation that those minimum scientific quality standards include open practices. By acknowledging that open practices should be considered by reviewers alongside other research norms, reviewers can collectively bring about a radical positive change in the culture of science.

## 2. The Peer Reviewers' Openness Initiative

Although some progress has been made towards open practices, actual behaviour in many scientific fields is far from the ideal of openness by default. We suggest a new strategy: a concrete statement of open-research requirements for publication, along with a plan for putting these into action. The Peer Reviewers' Openness Initiative (http://opennessinitiative.org) is a statement that researchers can sign that indicates that after a set, future date—1 January 2017—they will begin to apply certain minimal open research standards to the manuscripts they review. Central to the Initiative is the idea that reviewers can engage authors on issues of scientific openness during the review process.

### 2.1 Author-focused engagement

The reviewer/author relationship is at the heart of the peer-review process. At times, reviewers receive manuscripts for review that appear incomplete, such as missing a figure or missing statistical results. Before submitting a review on an incomplete manuscript, it is best to contact the authors through an appropriate channel for clarification on those incomplete aspects of the manuscript. Oversights can be easily corrected through constructive engagement with the authors.

We believe that a manuscript presenting work that is not sufficiently open should be treated as incomplete, warranting a similar strategy. We suggest that reviewers ask the authors through an appropriate channel whether their data and materials are open, and if not, for justification (see also [18]). Engagement with the authors should occur as soon as possible after the review assignment is accepted, in order to prevent unnecessary delays in the review process. This could be accomplished by the reviewer sending a simple email through the editor asking the authors about the openness of their manuscript.

The authors can respond in one of two ways to such a request: first, they can provide a link to the data and materials and agree to share a link in the published version of the manuscript. However, as we discuss later, there are valid reasons for incomplete openness. In the case that the authors will not open aspects of their research, the authors can respond with a justification for this lack of openness, and include their justification in the manuscript's author note so that reviewers and future readers can understand the Authors' reasons for not releasing some part of the data or materials. If a link to the data and materials or a justification for lack of openness is provided, and if these will be public, then the review process can continue as normal. If the authors will neither release their data and materials nor publicly justify their choice not to, then the reviewer should offer a short review that focuses only on the lack of openness and failure to justify it. We believe there is simply no argument for authors not justifying their data and material sharing practices; knowing why authors choose not to be open would be tremendously useful to the scientific community at large.

### 2.2 The initiative

Openness and transparency are core values of science. As a manifestation of those values, a minimum requirement for publication of any scientific results must be the public submission of materials used in generating those results. As reviewers, it is our responsibility to ensure that publications meet certain minimum quality standards. We therefore agree that as reviewers, starting 1 January 2017, we will not offer comprehensive review for, nor recommend the publication of, any manuscript that does not meet the following minimum requirements. Once such a manuscript has been certified by the authors to meet these minimum requirements, we will proceed with a more comprehensive review of the manuscript.

— *Data should be made publicly available.* All data needed for evaluation and reproduction of the published research should be made publicly available, online, hosted by a reliable third party.
— *Stimuli and materials should be made publicly available.* Stimulus materials, experimental instructions and programmes, survey questions and other similar materials should be made publicly available, hosted by a reliable third party.
— *In case some data or materials are not open, clear reasons (e.g. legal, ethical constraints, or severe impracticality) should be given.* These reasons should be outlined in the manuscript.
— *Documents containing details for interpreting any files or code, and how to compile and run any software programs should be made available* with the above items. In addition, licensing or other restrictions on their use should be made clear.
— *The location of all of these files should be advertised in the manuscript*, and all files should be hosted by a reliable third party. The choice of online file hosting should be made to maximize the probability that the files will be accessible for many years, and to minimize the probability that they will be lost for trivial reasons (e.g. accidental deletions, moving files).

Authors of submitted articles wishing to signal to reviewers and readers their adherence should add a statement to the author note specifying the data and materials that are open and where they can be found, or their justification for not sharing some of their data and materials.

## 3. Discussion

The idea of open practices being a minimal standard for research is not new. Peng *et al.* [9] suggested that open practices be a minimum requirement for epidemiology research. The core principles of UPSIDE highlight the public availability of data and materials [19]. Nosek *et al.* [20] broadly suggested the use of checklists by authors, reviewers and editors to help assess the overall quality of manuscripts, and Aleksic *et al.* [21] offer a general ‘open science peer reviewer's oath’ that suggests general principles for reviewers to help improve the status quo. The journal *Nature Genetics* has specified that all articles submitted must make available materials to reviewers and future readers, via permanent, public repositories [22]. *Nature Genetics* makes the very strong statement that they ‘see no reason to make exceptions or concessions to review or publish research articles—from any part of the world—that lack the most basic access to data’.

None of these other calls for more openness conflict with the Initiative, and we endorse them wholeheartedly. What is different about the Initiative is that it offers a specific, concrete mechanism—reviewers withholding comprehensive review—to achieve the goal of creating the expectation of open practices as a matter of scientific principle. The Initiative aims to solve the current dilemma facing researchers by taking advantage of the fact that openness is already valued by many in the scientific community [23]. Reviewers have several incentives to join the Initiative: it allows them to make their values manifest, without placing them at a disadvantage relative to one's peers and it temporarily eases their review load, because many papers will not meet the standards initially. Authors will obviously have the incentive to meet the requirements of the Initiative, because it will allow them to be published more quickly as reviewers will naturally allocate their limited time towards reviewing work that adheres to basic standards of scientific quality.

The Peer Reviewers' Openness Initiative website not only provides a way for researchers to voice their support for the Initiative and useful guidelines for signatories; it also provides links to resources that will help authors comply with the Initiative's guidelines. These resources are critical, because the intent of the Initiative is not to punish researchers who do not comply, but rather to increase the prevalence of open practices. The Initiative is not designed to be an onerous hurdle to publication, but is rather a gateway to a higher quality publication and a more efficient science.

## 4. Possible concerns

*My current practices are not in line with the Initiative. Can I sign the Initiative?* We suspect that there are many researchers who agree in principle with the goals of the Initiative, and wish that both their work, and the culture of science at large, were more open. Just because researchers are steeped in less-than-open traditions and practices that have been normative for many decades does not mean they cannot advocate for positive change for the field and for themselves. We suggest that those who resonate with the values of the Initiative sign it, with the dual intent of aiding the emergence of open practices through the peer review process and learning open practices.

*The Initiative overrides journal policy.* While some journals—for example, the Public Library of Science and the Royal Society journals^1^ —have policies requiring or encouraging open practices, most journals do not. It is our view that open research is not primarily a matter of policy; it is, rather, a matter of scientific values and the ultimate quality of the product. A policy merely reflects the values and the desire for quality. As with proper scientific attribution, openness is something that reviewers should address. If a scientific manuscript does not give credit where it is due, reviewers take a dim view of the quality of that manuscript. Even if a journal abandoned its attribution policies, reviewers would still have the right and responsibility to request proper attribution practices. Likewise, even in the absence of a journal policy of openness—or when a journal is not enforcing existing policy—reviewers have the right and responsibility to address open practices. We applaud journals and granting agencies that adopt open policies; obviously, science will work best when researchers' values and institutional policies are aligned. However, scientific quality is a question primarily for peers' consideration, and thus openness should be judged by peer reviewers.

*Open practices are not standardized.* Currently, there are a wide range of practices that might be described as open, from releasing data in an article's supplemental material to complete transparency as data are collected (e.g. [26]). Consensus has yet to be reached on what sort of openness is best for most scientific situations, and a request to authors to open their research could be interpreted in a number of ways. Certainly standardized open practices are superior to non-standardized open practices, but if open practices are not widespread, there is no reason for standardization. The demand for standardization must be driven by practice, rather than by an abstract desire to standardize that which is now occurring only rarely.

*Authors may lack training in open practices.* Acquiring new skills and knowledge is part and parcel of being a scientist. Academic researchers are life-long learners; in fact, academic research is predicated on the idea that we, as researchers, are constantly learning and sharing what we learn with others. Surely researchers that have the capacity to work at the boundaries of human knowledge also have the capacity to learn basic open research practices. We offer numerous examples on the Initiative website to help get researchers started; many more examples and tutorials can be found on the Internet.

Broadly speaking, effortless openness is a matter of forming good habits (e.g. [27–29]). The effort that openness takes is greatly diminished when researchers deliberately prepare to share their data with colleagues during the research process itself. At present, formal education in good research data management habits is typically not a component of scientific training [30]. We believe that future methods training in both the classroom and the laboratory should be focused on creating these good habits from the very first time a student encounters data. Good habits ensure that when a paper is submitted, no additional effort needs to be expended in preparing the data to be shared.

One thing that senior researchers can do to help students is to ensure that their data and research materials are well curated. In particular, having a laboratory-wide plan based on good research management practices—and making sure students understand it—has the immediate benefit that others in the same laboratory easily make use of the materials after the student has left, and the side benefit that openness will be a simple matter of making the already well-curated materials publicly available.

*Authors may be unable to share (some of their) data or materials.* It is our goal to create the strong expectation that research practices be open. In some cases, it may not be possible for data or materials to be shared for a variety of reasons, such as legal or ethical considerations. These cases can be dealt with on an individual basis, and the reasons for exceptions to open research practices should be clearly explained. Certainly, if anyone understands the constraints on openness that researchers face in a particular field, it will be the peer reviewers; this is a major point in favour of peer reviewers taking the lead on this issue.

The Royal Society Science Policy Centre [3] notes that in some cases, publicly releasing data can be ethically problematic. For instance, the rarity of some genetic disorders, such as Huntington's disease, makes the cases possibly identifiable. Research participants who consented to data release among qualified researchers may not consent to public data release. For some specialized research, secure data repositories accessible only by vetted researchers may be a better option than fully open data. Authors and reviewers should weigh the legal and ethical consequences of releasing data in the context of their research.^2^ Other problematic cases include issues of copyright: if scientists are using materials that are copyrighted by another organization, they may not have permission to release them publicly. The common thread in all examples of limited openness is that they should be thoughtful, justifiable in principle, and actually justified to readers and reviewers. For further discussion of the limits of openness, see [3, ch. 3].

Finally, some authors simply may not wish to share their materials publicly before acceptance of their paper. In such cases, some data repositories (e.g. Data Dryad and figshare) allow the embargoing of shared materials until publication, while still privately sharing the materials with reviewers. Such solutions remove from the authors the burden of remembering to share the materials after publication.

*As a reviewer, I do not have time to check Authors' analyses.* An important aspect of the Initiative is that it is not designed, by itself, as a fraud or mistake detection mechanism. It is designed, rather, to increase open practices. By joining the Initiative, you are not obligating yourself to check the analyses using the data provided. Reviewing remains as it always has been, except that you would have the option to check the analyses, if you chose to do so. We expect that once providing open data and materials becomes more common, norms will be established regarding how reviewers are expected to use these new resources, but until then reviewers should use their own judgment.

*I am concerned that the Initiative may be used aggressively.* The purpose of the Initiative is twofold: first, to alert reviewers to the fact that open practices are a legitimate target for peer review, and second, to give authors the incentive they need to begin implementing open research practices. The Initiative is thus intended entirely constructively. In some rare cases—for instance, in contentious areas of research—over-zealous application of the Initiative's standards might be used as a weapon by one's theoretical rivals. This would be undesirable, possibly causing researchers to resent requests for more openness instead of seeing them as opportunity to improve their practices. If any dispute arises over whether the points of the Initiative have been met, reviewers are encouraged to cede to the opinion of the authors and action editors.

Reviewers should assume good faith on the part of authors who have certified that they meet the points of the Initiative, and avoid aggressive, legalistic interpretations of the Initiative and making unreasonable demands. Signers of the Initiative should remember that the minimal requirement of the Initiative is mere public justification of lack of openness; even if one believes a particular justification is insufficient, if the justification is public it will become part of the broader conversation about openness among scientists. This broader conversation is substantially more valuable than openness in any single paper.

*How do I apply the terms as an action editor?* The application of the terms of the Initiative may complicate action editing if the Initiative is seen as too radical by journal senior editors. For this reason, the Initiative is targeted at reviewers and not action editors. Signatories of the Initiative are, of course, free to make reference to the Initiative as action editors, but signing does not obligate them to do so. As a signatory of the Initiative, your role as an action editor and your role as a reviewer are seen as separate. There is one important role that action editors have as a part of their typical responsibilities: judging whether authors have made a good-faith effort to meet a reviewer's request for open practices. As noted above, we believe that reviewers should defer to Authors' and action editors' opinions about whether an article has met the requirements of the Initiative. One of the main responsibilities in action editing is weighing the concerns of reviewers and authors. We expect that action editors will not find juggling demands for open research to be any more problematic than juggling other reviewer demands.

## 5. Conclusion

The Peer Reviewers' Openness Initiative provides the best possible chance for open research practices to become commonplace in the scientific literature. Now is the time for all of the sciences to be open; we hope that researchers who value open research practices—even ones who have not yet put them into action—will join us in signing the Initiative and help promote open research. We must let current and future researchers know that openness is not just a value we talk about, but one we make manifest.

## Footnotes

1 The Public Library of Science does not require, but ‘strongly recommends’ public deposit of data and materials [24], while the Royal Society requires public deposit of data but does not have requirements for materials [25]. We are not aware of any journals that require public justification where openness is lacking; we believe this is key.

2 For authors that would like to approach their organization's ethics review board about open data issues, the Initiative website contains a form letter that might be helpful: https://opennessinitiative.org/enlisting-help-from-your-institution/.
